# Montreal Veterinary Medical Association

**Published:** 1893-01

**Authors:** 


*Montreal Veterinary Medical Association.* The meeting of the Montreal Veterinary Medical Association was held November 24th. The meeting was called to order with Vice-President Dr. Baker in the chair. After the reading of the minutes of the previous meeting, Dr. Baker made a few very appropriate remarks as to the sad death of one of the members of the society, Mr. A. J. A. Ewing, class of '93.

After some further business, Mr. Wylie was called upon to read a paper on the "Treatment of Flatulent Colic." The case reported was of an aggravated form of six hours' standing. He used hypodermic injection of:

Eserine Sulphate, 2 grains;
Pilocarpine Muriate, 1 grain;
Aquæ ad, one and one-half drachms, with a drench of Chloral Hydrate one and one-half ounces.
Aquæ ad, 1 pint.

Flatus began to pass in sixteen minutes; the bowels moved in thirty-five minutes, and at the expiration of two hours had had nine operations of bowels and was reduced to normal size. During the summer he used it on twenty-one cases of flatulent and six cases of spasmodic colic without a fatal result.

During the discussion Dr. Baker stated that he had not found flatulent colic a very fatal disease, and that the simplest remedies were often the best and for his purpose bicarb. soda given every fifteen minutes with hard rubbing were most satisfactory.

Mr. McGuire was then called upon to read his paper on "Glanders and Farcy," of which the following is a synopsis:

He described the nature of the disease, animals affected, horse being most largely, and it runs a variable course until it produces death. Parts of the body where it is localized and complications which may go hand in hand with it; the theories as to its origin and spread. It occurs under four forms: acute glanders, chronic glanders, acute farcy and chronic farcy. As to the history of the disease, it can be traced back as far as time of Constantine the Great. At this early period they described glanders nearly same as we have it to do, with views differing a little as to its spreading. It was introduced into America in latter part of 18th century and spread with great rapidity. Taking up the etiology, the direct cause is contagion, although may have several predisposing causes. The disease does not affect all animals alike, although it is a general constitutional malady. An animal may be inoculated in various ways, just so the virus gains admittance to the blood. An animal may be inoculated and retain the virus in its system for a great length of time, and at the same time defy the most skillful veterinarians to diagnose it.

Comparing virus of acute and chronic glanders, one may produce the other or reproduce itself, although the discharge from chronic glanders is not so apt to produce the disease. The disease seems to assume a more acute form in high bred animals than in lower grades, which is usually of a chronic character. In dog the disease appears as an ulcer at point of inoculation, which may heal and leave the animal healthy. Man is more susceptible than dog; it may assume either a local or constitutional form. The cat is susceptible and infects system from point of inoculation. Goat is very susceptible and may develop from ingestion of infected articles. Pig, horned cattle and barnyard fowls seem to be exempt. The predisposing causes are those that would not be in accordance with sanitary principles, or some debilitating disease; the most common forerunner is diabetes incipidus.

Prof. Williams' views on glanders and farcy were brought forth, and the possibility of recovery from farcy has led some to consider them separate diseases. The period of incubation is short; the disease shows itself in from three to six days after inoculation, or it may extend to six weeks. Symptoms—Formation of tubercles in the respiratory tract, accompanied by discharge and cough; appearance of farcy buttons, which form ulcers; enlargement of lymphatics; swelling of sub-maxillary glands; bleeding from nose; sudden swelling of hind leg; difficulty in respiration; temperature rises, and appears as if suffering from acute pneumonia, but is no dullness on percussion. The symptoms of chronic glanders and farcy are same but do not come on so rapidly. In all animals have loss of flesh, become hide-bound and emaciated.

*Post Mortem.* Mucous membrane of nasal passages influenced and studded with pustules and ulcers, which discharge a saniou· fluid. Bones of nasal passages may be necrosed. Submaxillary glands congested. Lungs may be studded with nodules and infiltrated, which vary in size and structure, resembling tuberculosis. May see dull grey spots in liver and spleen.

For prevention and suppression he recommends the destruction of all animals known to be affected. Isolation of those suspected or exposed. Thorough cleansing of the buildings previously occupied and then disinfecting. Several methods for disinfecting were set forth. The most practical was by means of sulphurous acid, from burning sulphur. After being thoroughly disinfected and cleaned, they should be open for access to free air. All wearing apparel and harness should be treated in a like manner. In some instances this will not be practicable, as out-buildings are of a poor order, and then it is best to consign them to the flames.

The experiments with mallein for diagnosis of "Glanders" were received, and the discovery of this method of diagnosing occult cases was pronounced as one of the most valuable of recent medical methods.

Prof. D. McEachran intimated that arrangements had been made for supplies of both "mallein" and "tuberculin" to be made use of in the practice of the college.

